# 

**Published:** 1894-02-10

**Authors:** 


**Notice to Advertisers and Subscribers.**

All Advertisements, Orders for Copies of The Hospital, and Business Communications should be addressed to The Publisher, *not to the Editor*, The Hospital, 428, Strand, W.C.

The Cost of Subscription, post free, is as follows:—Per week, 2½d.; per quarter, 2s. 8d.; per half-year, 5s. 3d.; per annum, 10s. 6d. Foreign:—Per Annum, 12s. 8d.; payable, in all cases, in advance.

**OUR NEW OFFICES.**

428, Strand, W.C., will henceforth be the Office of this Journal.

**Notices of Births, Deaths, and Marriages** are inserted in this column at a charge of 2s. 6d. for an announcement not exceeding four lines.

**NOTICE TO CORRESPONDENTS.**

All MS., Letters, Books for Review, and other matters intended for the Editor should be addressed The Editor, The Lodge, Porchester Square, London, W.

The Editor cannot undertake to return rejected MS., even when accompanied by stamped directed envelope.

**"Burdett's Hospital Annual," 1893.**

Fourth year of publication. 700 pp. Boards, gilt lettering. A most complete guide to British, Colonial, Indian, and American Hospitals, Dispensaries, Nursing and Convalescent Institutions, and Asylums. Price 5s. Published by The Scientific Press (Limited), 428, Strand, W.C.

**NOTICE.—The Index for Vol. XIV. (ending Sept. 30th, 1893) is on sale at the Office of this Journal, price 2½d., post free.**

### SCRAPS AND GLEANINGS.

Dr. Thomas Pugh, it has just been announced, has endowed the Medical Benevolent College with the munificent bequest of £5,000.

The annual meeting of the Londonderry City and County Infirmary was held recently. A balance to the good of £55 remains on the statement of accounts.

Notwithstanding a year of commercial depression, the committee of the Tiverton Infirmary and Dispensary give a fairly satisfactory report at the annual meeting.

The Committee of the Convalescent Home for Women and Children, New Brighton, are urgently soliciting for subscriptions towards the £500 required to furnish the new wing.

A donation of £50 has been made to the funds of the above institution. This gift was forwarded through the secretary, with the request that the donor's name was not made public.

At a meeting of the representatives of the mills and workshops in connection with the Preston and County Royal Infirmary, the committee reported a decrease of £95 in the amount collected.

Throughout the year sixty-four small patients have been benefited at the Convalescent Home for Poor Children at St. Leonards. Contributions amounting to £614 have been forwarded towards the funds of this charity.

A meeting of the council of the Metropolitan Hospital Sunday Fund was held at the Mansion House on February 2nd, with the Lord Mayor in the chair. The proceedings were of a formal character, and occupied only a short time.

A new mortuary is being added to the buildings of the Royal Albert Edward Infirmary, Wigan. The committee contemplate erecting better and more suitable rooms for the nurses, which at present are singularly inadequate.

By direction of the Princess of Wales, the sum of £9 1s. 8d. has been forwarded to the secretary of the British Home for Incurables, being a further donation from the proceeds of the sale of Canon Fleming's sermon—"Recognition in Eternity."

The subscriptions towards the building fund of the Royal Alexandra Hospital, Rhyl, at present amount to £8,331, besides a balance in hand of £1,065. There is a further sum of £106, which was collected by Dr. Lloyd on behalf of the accident ward.

During the year 633 patients have received assistance from the Stockton Dispensary. The treasurer's report for the twelve months under review stated that the payments for that time had amounted to £187, and that £14 was now due to the bank.

The improvements in the City of Dublin Hospital, amounting almost to the erection of a structure double the size of the old building, are now fast approaching completion, and in three months' time, presumably, the renewed hospital will be fully equipped.

The ordinary income of the Glasgow Eye Infirmray amounted in 1893 to £3,740, and the ordinary expenditure to £3,677. Legacies amounting in value to £2,233 were received during that same period; whilst contributions from public works had brought in £1,920.

The result of last year's working of the Royal Bath Hospital, Harrogate, has been in all respects satisfactory. There has again been a gratifying increase in the amount of subscriptions. Both the annual subscriptions and the number of patients are greatly in excess of any previous year.

Considerably over £3,000 has been contributed towards the Newcastle Throat and Ear Hospital during the last ten years by the working classes. There is a plan on foot to erect new premises in place of those already existing, which have proved inadequate to meet the growing needs of this useful institution.

Some idea of the magnitude of the work which the St. John Ambulance Association is doing, owing to the help generously given by the medical profession, may be gathered from the fact that it issued in the twelve months ending Michaelmas, 1893, no fewer than 30,761 first-aid and nursing certificates.

At an extraordinary meeting recently called of the Guildford and Godalming Joint Hospital Board it was decided that the Local Government Board should be applied to for powers to borrow £8,000 for the purchase of the hospital, and for carrying out the improvements and additions considered necessary.

The committee of the Andover Cottage Hospital, in submitting their 17th annual report, are able to state that the hospital continues to go on satisfactorily, notwithstanding some difficulties, the chief one being insufficient income. The year began with a deficiency of £100, but has now been considerably reduced.

The 117th annual report of the Dumfries and Galloway Royal Infirmary for the year ended has just been issued. The Treasurer's accounts show that the ordinary expenditure has exceeded the ordinary income by £177. But the income for the year has not only wiped off this deficit, but has added £855 to the capital of the institution.

The committee of the Sussex Eye Hospital at Brighton are calling attention to the alarming financial position of the institution at the close of the year, and a serious deficit on the balance sheet was to be faced. Unless pecuniary assistance is forthcoming further retrenchments will have to be devised, which will necessarily much cripple the usefulness of the hospital.

The committee of the West Kent General Hospital, Maidstone, in presenting their annual report, remark upon the success which has attended the past year's working of the institution. The hospital is managed in a way which does credit to all concerned, and the energy displayed by the committee has met with an equal response in the interest shown in the surrounding district.

In submitting the balance-sheet for the past year of the Peterborough Infirmary, the treasurer reported that a deficit of £362 existed on the accounts of the institution. Further, it was also pointed out, that the question had very seriously arisen of closing the infirmary fever house, the present building not only being inadequate, but its proximity to the general wards was a constant source of danger.

### BOOKS RECEIVED.

H. K. LEWIS.

"Headache, and Other Morbid Cephalic Sensations." By Harry Campbell, M.D.

"Common Neuroses." By James F. Goodhart, M.D. Second edition.

A. CONSTABLE, EDINBURGH.

"Atlas of Clinical Medicine." By Byrom Bramwell, M.D. Vol. II. Part III.

L. N. FOWLER and Co.

"The Child, Physically and Mentally." By Bertha Meyer. 2s.

"Health, and the Various Methods of Cure." By J. H. Rausse.

H. GREVEL and Co., LONDON.

"Thus Shalt Thou Live." By Sebastian Kneipp. Translated from nineteenth German edition.

MACMILLAN and Co.

"Letters of Travel." By Phillips Brooks, late Bishop of Massachusetts.

HENRY KIMPTON.

"Modern Gynæcology." By Charles H. Bushong, M.D.

**Pamphlets, Periodicals, &c.**—Reichs; Medicinal Auzliger; Cassell's Magazine; Quarterly Paper; Edinburgh Medical Missionary Society; The Child's Guardian; The Zenana; Amateur Gardening; Service of the King.

### MEDICAL VACANCIES AND APPOINTMENTS.

### APPOINTMENTS.

C. J. Acton, M.R.C.S., L.R.C.P., D.P.H., appointed Medical Officer and Public Vaccinator of the Eighth District, Blything Union. F. A. Baldwin, L.S.A., appointed Medical Officer to the Seventh District of St. Saviour's Union, Walworth. Dr. Beaman, appointed Medical Officer for the Misterton District of the Gainsborough Union. C. G. Brown, L.R.C.P.Edin., M.R.C.S.Eng., appointed Honorary Medical Officer to the Goole Cottage Hospital. G. H. Butler, L.R.C.P.I., L.R.C.S.Edin., appointed Medical Officer for the Wealdstone District of the Hendon Union. W. W. Cheyne, M.B., F.R.C.S., appointed Consulting Surgeon to the North London Hospital for Consumption, Hampstead and London. S. B. De Butts, M.D.Brux., M.R.C.S.Eng., &c., appointed Anæsthetist at the London Lock Hospital, Dean Street, W. F. G. Englebach, L.R.C.P.Lond., M.R.C.S.Eng., appointed Medical Officer for the Newtonhampstead District of the Newton Abbott Union. E. C. Fischer, M.B., C.M.Edin., appointed House-Surgeon to the Royal Ophthalmic Hospital, Moorfields, E.C. H. Fitzgibbon, M.D., T.C.D., Medical Officer to the General Post Office, Dublin, appointed Secretary in Ireland to the British Postal Medical Officers' Association. W. D. Gimson, M.R.C.S., L.R.C.P.Lond., appointed Medical Officer for the Third District of the Chelmsford Union, J. F. Gordon, M.D.R.U.I., appointed Medical Officer of the Branch Workhouse at Maghull of the Parish of Liverpool. E. Lloyd-Williams, M.R.C.S., L.R.C.P., L.S.A., L.D.S., appointed Dental Surgeon to the Dental Hospital of London. A. McGillivray, C.M., M.B.Aberd., appointed Joint Ophthalmic Surgeon to the Dundee Royal Infirmary. E. D. Marriott, L.R.C.P.Edin., L.F.P.S.Glasg., appointed Medical Officer for the No. 5 District of the Nottingham Union. R. L. Meade-King, M.B., B.S.Durham, M.R.C.S., L.R.C.P., appointed Assistant House-Surgeon to the Devon and Exeter Hospital. Mr. D. Muir, appointed Medical Officer for the Holmworth District of the Highworth and Swindon Union. T. Newby, M.D.St.And., M.R.C.S.Eng., reappointed Port Medical Officer of the Grimsby Town Council. F. W. Ramsay, M.D.Dunelm, M.S., F.R.C.S.Edin., appointed Honorary Surgeon to the Royal Victoria Hospital, Bournemouth. H. B. Robinson, M.S., M.D.Lond., F.R.C.S., appointed Assistant Surgeon to St. Thomas's Hospital. W. J. Stephens, M.R.C.S.Eng., L.R.C.P.Lond., appointed Second Assistant Resident Surgeon to the General Dispensary, Nottingham.

### VACANCIES.

### Resident Medical Officers.

Alnwick Infirmary. House Surgeon, unmarried. Salary £120 per annum, with furnished apartments, attendance, coals, and gas. Applications to W. T. Hindmarsh, Honorary Secretary, 26, Bondgate Without, Alnwick, by February 16th.

Oxford Eye Hospital. House Surgeon. Appointment for one year. Salary £50, with board and lodging. Applications to Mr. B. H. Baden-Powell, Honorary Secretary, 29, Banbury Road, Oxford, by February 24th.

Royal Surrey County Hospital, Guildford. House Surgeon. Salary £80 per annum, with board, lodging, and laundry. Applications to the Honorary Secretary by March 10th.

Salford Royal Hospital. House Surgeon, doubly qualified. Salary £100 per annum, with board and residence. The Junior House Surgeon is a candidate, and in the event of his being appointed the post of Junior House Surgeon will be vacant. Salary £50 per annum, with board and residence. Applications to the Secretary by February 15th.

Tiverton Infirmary and Dispensary. House Surgeon and Dispenser. On the Medical Register for Great Britain and Ireland. Salary £100 per annum, lodgings, attendance, fire, and light. Application to the Secretary by February 23rd.

### Non-Resident Medical Officers.

Dental Hospital of London, Leicester Square, W.C. Assistant Dental Surgeon. Applications to J. Francis Pink, Secretary, by March 12th.

Kilburn, Maida Vale, and St. John's Wood Dispensary. Vacancy on the Honorary Medical Staff. Applications to the Secretary, 13, Kilburn Park road, by February 14th.

Liverpool Hospital for Cancer and Skin Diseases. Honorary Assistant Surgeon. Applications to Mr. A. N. Talbot, 3, Rumford Street, Liverpool, by February 20th.

Manchester Institution for Diseases of the Ear. Honorary Assistant Surgeon. Applications to the Honorary Secretary, Mr. T. C. P. Gibbons, 33, Mosley Street, Manchester, by February 17th.

New Hospital for Women, 144, Euston Road, N.W.—Lady Dispenser. Salary, £90 per annum. Applications to the Secretary, by February 17th.

Owens College, Manchester. Professor of Zoology. Applications to the Council of the College, under cover to the Registrar, by April 3rd.

Parish of St. Leonard, Shoreditch.—Medical Officer of Health. Salary £500 per annum. Must reside within one mile from the boundary of the parish. Applications, on forms to be obtained of the Clerk, marked "Medical Officership," to be sent to H. Mansfield Johnson, Solicitor and Clerk, Shoreditch Town Hall, Old Street, E.C., by February 13th.

St. George's and St. James's Dispensary, 60, King Street, Regent Street, W, Physician. Applications to St. Leger Bunnett, Secretary, by February 14th.

**SALFORD ROYAL HOSPITAL.**

